# Soluble CD27 is an intrathecal biomarker of T-cell-mediated lesion activity in multiple sclerosis

**DOI:** 10.1186/s12974-024-03077-9

**Published:** 2024-04-12

**Authors:** Maria T. Cencioni, Roberta Magliozzi, Ilaria Palmisano, Keittisak Suwan, Antonella Mensi, Laura Fuentes-Font, Luisa M. Villar, José I. Fernández-Velasco, Noelia Villarrubia Migallón, Lucienne Costa-Frossard, Enric Monreal, Rehiana Ali, Marina Romozzi, Nicholas Mazarakis, Richard Reynolds, Richard Nicholas, Paolo A. Muraro

**Affiliations:** 1 Department of Brain Sciences, Imperial College London, Du Cane Road 160, London, W12 0NN UK; 2 Department of Biotechnology, University of Verona, Verona, Italy; 3 Department of Neuroscience, Department of plastic and reconstructive surgery, The Ohio State University College of Medicine, Columbus, OH US; 4 Department of Immunology, Hospital Universitario Ramón y Cajal, REEM, IRYCIS, Madrid, Spain; 5 Department of Neurology, Hospital Universitario Ramón y Cajal, Madrid, Spain; 6 Department of Neuroscience, Universita’Cattolica del Sacro Cuore, Rome, Italy; 7 Department of Neuroscience, Organi di Senso e Torace, Fondazione Policlinico Universtario Agostino Gemelli IRCCS, Rome, Italy

## Abstract

**Objective:**

Soluble CD27 is a promising cerebrospinal fluid inflammatory biomarker in multiple sclerosis. In this study, we investigate relevant immune and neuro-pathological features of soluble CD27 in multiple sclerosis.

**Methods:**

Protein levels of soluble CD27 were correlated to inflammatory cell subpopulations and inflammatory cytokines and chemokines detected in cerebrospinal fluid of 137 patients with multiple sclerosis and 47 patients with inflammatory and non-inflammatory neurological disease from three independent cohorts. Production of soluble CD27 was investigated in cell cultures of activated T and B cells and CD27-knockout T cells. In a study including matched cerebrospinal fluid and post-mortem brain tissues of patients with multiple sclerosis and control cases, levels of soluble CD27 were correlated with perivascular and meningeal infiltrates and with neuropathological features.

**Results:**

We demonstrate that soluble CD27 favours the differentiation of interferon-γ-producing T cells and is released through a secretory mechanism activated by TCR engagement and regulated by neutral sphingomyelinase. We also show that the levels of soluble CD27 correlate with the representation of inflammatory T cell subsets in the CSF of patients with relapsing-remitting multiple sclerosis and with the magnitude of perivascular and meningeal CD27 + CD4 + and CD8 + T cell infiltrates in post-mortem central nervous system tissue, defining a subgroup of patients with extensive active inflammatory lesions.

**Interpretation:**

Our results demonstrate that soluble CD27 is a biomarker of disease activity, potentially informative for personalized treatment and monitoring of treatment outcomes.

**Supplementary Information:**

The online version contains supplementary material available at 10.1186/s12974-024-03077-9.

## Introduction

Multiple sclerosis (MS) is an immune-mediated disease of the central nervous system (CNS) characterized by inflammation, demyelination and neurodegeneration [1, 2]. Intrathecal inflammation with mild lymphocytic pleocytosis and oligoclonal immunoglobulins is routinely detectable [3, 4] and is consistent with persistent activation of B and T cells in the CNS [5–8]. Management of MS would benefit from biomarkers that reflect the pathological processes of the disease and enable disease monitoring and stratified or ideally personalized treatment in individual patients [9, 10].

Soluble CD27 (sCD27) has been detected in the cerebrospinal fluid (CSF) of patients with neuroinflammatory diseases [11–13] and described as a prognostic biomarker of MS in clinically isolated syndrome (CIS) [14] and childhood-acquired demyelinating syndromes [15]. Furthermore, sCD27 was found to be the best single biomarker of active intrathecal T-cells in progressive MS [16, 17] as well as of B cells in patients with relapsing-remitting (RR)-MS [18] and was reported to be associated with a higher risk of developing new MRI lesions when present in the CSF at the time of diagnosis [19]. The cellular source and role of sCD27 in the pathogenesis of MS remain poorly understood, and a better understanding of the immune and neuropathological processes associated with the biomarker could provide valuable insights into disease mechanisms. Several studies have demonstrated that the soluble form of CD27 competes agonistically or antagonistically with its membrane-bound counterpart for ligands [20, 21] and contributes to T cell activation and proliferation suggesting its involvement in inflammation and immune response [22]. We hypothesised that abnormally high levels of sCD27 promote the differentiation of inflammatory T cells in patients with MS. By analysing CSF from different patient cohorts, we demonstrate that CSF levels of sCD27 are significantly higher in patients with RR-MS compared to other inflammatory and non-inflammatory neurological diseases (OND). With studies in vitro, we show that sCD27 contributes to the differentiation of IFN-γ-producing-T cells and that high levels of sCD27 are released mainly by T cells upon TCR activation through an exocytosis mechanism regulated by neutral sphingomyelinase (nSMase). Furthermore, with immunopathological studies of paired CSF and brain tissues obtained from post-mortem MS cases, we demonstrate a correlation between levels of sCD27 in the CSF and the number of CD27 + T cells in perivascular and meningeal areas, most prominently found in cases with early active MS lesions and an overall high degree of lesion activity. Our results suggest that sCD27 is a biomarker of T cell-mediated inflammatory disease activity in patients with MS and explain mechanistically its association with neuropathological processes that strongly affect disease outcomes.

## Materials and methods

### CSF and blood samples from participants

The National Health Service Research Ethics Committee had approved this study (IRAS ID: 204,872 and FIS PI15/00513), and all subjects have provided written informed consent to participate in the study.

CSF supernatants were collected and centrifuged at 335 g for 10 min at 4^0^ C to separate the cell pellet from the supernatant according to the standard procedures [16].

In this study, we analysed three distinct cohorts of CSF samples, including samples from individuals with MS and control subjects.

The first cohort included matched serum and CSF samples collected from 65 patients with RR-MS and 35 with OND in Hospital Universitario Ramon y Cajal of Madrid. The demographic and clinical data of MS and OND were reported in Supplementary Table 1.

Serum and CSF supernatants were analysed for the protein levels of sCD27. For surface antigen identification, cellular pellets obtained after CSF centrifugation were resuspended in their residual volumes, stained with appropriate amounts of monoclonal antibodies (according to manufacturer’s instructions) for 30 min at 4 °C in the dark, washed twice with phosphate-buffered saline (PBS), and analysed in a FACSCanto II flow cytometer (BD Biosciences).

For intracellular cytokine detection, cellular pellets were incubated for 4 h at 37 °C in 5% CO2 with 50 ng/ml Phorbol 12-myristate 13-acetate (PMA, Sigma-Aldrich, St. Louis, MO) and 750 ng/ml Ionomycin (Sigma-Aldrich) in presence of 2 µg/ml Brefeldin A (GolgiPlug, BD Biosciences) and 2.1 µM Monensin (Golgi Stop, BD Biosciences). After incubation, cells were washed once and stained with the appropriate amounts of monoclonal antibodies (according to the manufacturer’s instructions) for 30 min at 4 °C in the dark. Then, cells were washed once, fixed and permeabilized with Cytofix/Cytoperm Kit (BD Biosciences). After that, cells were washed twice and subjected to intracellular staining with monoclonal antibodies recognizing different cytokines, for 30 min at 4 °C in the dark. Finally, cells were washed and analysed in a FACSCanto II flow cytometer. The following monoclonal antibodies were used in the study: CD3-PerCP, CD3-BV421, CD5-APC, CD8-APC-H7, CD14-FITC, CD19-PE-Cy7, CD25-PE, CD27-FITC, CD38-PE-Cy5, CD45-V500, CD56-APC, CD127-BV421, IFNγ-FITC, TNF-PerCP-Cy5.5, and GM-CSF-PE (BD Biosciences, San Diego, CA), IL17-APC (R&D Systems, Minneapolis, MN). The different cell subsets were identified according to the combination of markers shown in supplementary Table 2.

The second and third cohorts include CSF from 71 patients with a diagnosis of RR-MS and SP-MS and 12 control donors with a diagnosis of headache, migraine, and idiopathic intracranial hypertension (IIH) (OND). The samples were collected from Charing Cross Hospital and only the CSF supernatants were available and investigated for the presence of inflammatory markers (cytokines and chemokines) and sCD27. The demographic and clinical data of those cohorts were reported in Supplementary Table 1.

### Enzyme-linked immunosorbent assay (ELISA)

ELISA was performed to investigate protein levels of sCD27, BAFF, CXCL13, levels of cytokines IFN-g, TNF (DuoSet Elisa development System, R&D, UK) and NFL-light (Tecan, Switzerland) according to the manufacturing protocols. Briefly, the flat bottom 96 plates were coated with a working solution of capture antibody and filled with a blocking solution of PBS with 1% BSA (bovine serum albumin, Sigma). Then, the plates were filled with 100 ul of diluted samples and 100 ul of a serial dilution of standard analyte. After 2 h of incubation, the plates were washed with PBS with 1% Tween 20 and incubated with 100 ul of a working solution of the detection antibody for 1 h. Then the plates were washed and 100 ul of working solution of streptavidin-HRP was added for 20 min. After washes, the substrate solution was added for 20 min, and a working solution of sulphuric acid (2 N H2SO4) was added to block the reaction. The plates were run in a microplate reader (Promega) set at 450 and 560 nm. The readings (absorbance) at 450 nm were corrected by subtraction of the readings at 560 nm. All the samples were analysed in duplicate.

### Electro chemiluminescent assay

Meso Scale Discovery detection system (MSD; Meso Scale diagnostics, UK) was performed to quantify the concentration of selected biomarkers in the CSF supernatants. The MSD U-Plex plates were customised with the cytokines (IL-6, IL-10, TNF) and chemokines (CCL19, CXCL10, CXCL12) and developed as suggested by the supply (Meso Scale diagnostics, UK). All the samples were analysed in duplicate.

#### Preparing multiplex coating solution

200 ul of biotinylated antibody for each analyte was mixed with 300 ul of assigned linker and incubated for 30 min a room temperature. Then, 200 ul of stop solution was added to each vial and left for another 30 min. 600 ul of each U-Plex coupled antibody solution was mixed in one tube and 2.4 ml of stop solution was added to bring the final volume to 6 ml.

#### Coating the U-Plex plate

A binding plate was coated by adding 50 ul of the multiplex coating solution to each well and incubated for one hour on the shaker a 200 rpm at room temperature. Then, the coating solution was aspirated, and the plate was washed 3 times with PBS-T (PBS with 10% of Tween 20).

#### Assay procedure

50 ul of each diluted sample and serial dilution of standard curve were added to the well and the plate was incubated for one hour at room temperature on the shaker at 200 rpm. Then, the plate was washed three times with PBS-T and 50ul of working solution of detection antibody was added to each well and incubated for one hour at room temperature on the shaker a 200 rpm. Then, the washing process was repeated and PBS-T and 150 ul of 2fold-concentrated read buffer were added for MSD analysis.

### Cell culture and protein extraction

Peripheral blood mononuclear cells (PBMCs) were isolated from fresh blood samples of 25 healthy donors by centrifugation on a gradient of Ficoll-plaque as described [23].

Briefly, 40 ml of whole blood was diluted with PBS and 35 ml of diluted blood was layered slowly on a gradient of 15 ml of Ficoll-plaque in 50 ml of falcon tube. The samples were centrifuged at 1800 rpm for 30 min with the break turned off. The layer of mononuclear cells was collected and washed twice with PBS through centrifugation at 1600 and 1400 rpm.

CD4, CD8 T and B cells were isolated from PBMCs using magnetic beads (CD4 + T cell isolation kit, CD8 + T cell isolation kit and CD19 Micro beads, Miltenyi Biotech, Germany) as suggested by the technical data sheet.

In a two-step procedure, CD4 + and CD8 + T cell isolation kits were used to isolate untouched CD4 + or CD8 + T cells. The PBMCs were stained with a cocktail of biotin monoclonal antibodies against CD4 + T cells (in CD8T cell isolation) or CD8 + T cells (in CD4 T cell isolation), monocytes, eosinophils, neutrophils, B cells, NK cells, γδ T cells, granulocytes, and erythroid cells in buffer containing PBS with 0.5% of FBS and 2 mM of Ethylenediaminetetraacetic (EDTA) for 5 min in ice. Then, CD4 + T or CD8 + T microbeads that are conjugated to anti-biotin antibody and monoclonal anti-CD61 antibody (in CD4 T cell isolation) were added to the cells and incubated for 10 min in ice. The cells were layered onto an LS column placed in a magnetic field previously activated with a rinsing buffer (PBS with 2 mM of EDTA). The column retains labelled cells and elutes unlabelled CD4 + or CD8 + T cells based on the isolation kit used for the separation.

The CD19 isolation kit is a one-step positive separation for isolating CD19 + B cells. First, the PBMCs were stained with microbeads conjugated with antibodies against CD19 B cells for 15 min in ice and then washed in a buffer at 300 x g for 10 min at 4 degrees. Then, the cell pellet was resuspended in 500 ul of buffer and layered onto an MS column previously placed in a magnetic field and activated with rinsing buffer. The column was washed with rinsing buffer to elute the negative fraction. The column was removed from the magnetic field and CD19 + B cells were collected by washing out the column with 1 ml of rinsing buffer. The cells were stained with fluorescent antibodies and their purity was evaluated by analysing them with Flow cytometry LSRII.

#### B cell activation

Isolated CD19 cells were suspended in a completed medium (RPMI-1640 medium completed with 10% of FBS) (Gibco, ThermoFisher Scientific, UK) and seeded in a round bottom 96-well plate at the concentration of 2 × 10^5^ cells/well and then stimulated with F(ab)2-goat anti-human IgMIgG, (H + L) functional grade (5ug/ml, eBioscience), CD40L (1ug/ml, functional grade, Invitrogen), CpG ODN 2006 (TLR9 ligand, 2.5ug/ml, InvivoGen) and resiquimod (R848, TLR7 and TLR8 ligand, 1ug/ml, Sigma-Aldrich). The cell culture supernatants were collected at 12, 24, and 48 h and analysed for the presence of sCD27 and TNF.

#### B cell activation for western blotting

Isolated CD19 B cells were suspended in a completed medium and seeded in a 48-well plate at the concentration of 5 × 10^5^ cells/well and then stimulated as reported above for 48 h.

The cell pellets were harvested and processed for protein extraction as reported below.

#### CD4 and CD8 T cell activation

Isolated CD4 + or CD8 + T cells were suspended in complete medium and seeded at the concentration of 2 × 10^5^ cells per well in a sterile round bottom 96-well plate pre-coated with 1, 5 and 10 ug/ml of anti-CD3 (clone UCHT1, Biosciences) and incubated a 37 °C for 72 and 96 h. Supernatants were collected at 72, and 96 h and analysed for protein levels of IFNg and sCD27. 1 × 10^6^ cells of purified CD4 and CD8 T cells were seeded on 48 well plates, pre-coated with 5ug/ml of aCD3 (clone UCHT1,eBiosciences) for 72 h. The cells were harvested and centrifuged at 1400 rpm. The cell pellets were processed for protein extraction and levels of sCD27 and CD27 have been analysed as reported below.

#### Protein extraction and quantification

Cell pellets were washed 2 times in PBS at 2,500 rpm for 10 min at 4 degrees and then resuspended in cell extraction buffer (RIPA buffer with added protease and phosphate inhibitor cocktail solution, Invitrogen, UK) for 30 min on the ice. Then, the extracts were centrifuged for 10 min at 16,000 rpm at 4 °C. The supernatants were collected and stored at -80 °C. Protein quantification was performed with BCA protein assay (Pierce) as suggested by the supply and 30 ug of protein was loaded for each condition either T or B cells.

### CRISPR/Cas9 RNPs

The CD27 gene ablation on CD4 T cells was performed as described [24].

#### T-cell activation

A sterile, non-treated 48-well tissue culture plate was coated with anti-CD3 antibody at the concentration of 10ug/ml. The pellet of isolated CD4 T cells was suspended in complete RPMI medium (high-glucose RPMI-1640, 10% FBS, penicillin-streptomycin 50ug/ml, sodium pyruvate 5mM, HEPES 5mM) with anti-CD28 (5ug/ml, clone CD28.2) and IL-2 (20U/ml) at the final concentration of 2,5 × 10^6^ cells/ml. 500 ul of cell suspension was added at each well of the anti-CD3 plate bound plate and incubated at 37 °C with 5%CO2 for cell stimulation and activation. After 48–72 h, the cells were collected, washed two times in PBS and suspended in PBS at the concentration of 2,5 × 10^6^ cells/ml. The volume of 2 × 10^5^ cells was centrifuged and the cell pellet was suspended in Neon R buffer before nucleoporation (Neon™ transfection system, Thermo Fisher scientific, UK).

#### crRNP generation

crRNPs were synthesized in vitro by incubation of 1ul of tracrRNA (160 μm) and 1ul of gRNA (160 μm) in a sterile PCR tube to form 2ul of complex gRNA: tracrRNA at the concentration of 80 μm. A mix of positive and negative controls was prepared at the same time. Then, the mixtures were incubated at 37 °C for 30 min in the thermocycler to promote the duplex formation. 2ul of Cas9-NLS protein was added to the RNA mixtures and then incubated at 37 °C for 15 min to form crRNPs.

#### crRNP nucleotrasfection

500 ul of fresh medium (high-glucose RPMI-1640, 10% FBS, sodium pyruvate 5mM, HEPES 5mM) with 20U/ml of IL-2 were added at 24-well plate.

For each nucleotransfection reaction, 4ul of crRNPs were added to the cell suspension prepared in R buffer. Then, 10 ul of the cells/crRNP mixture were transferred into 10ul Neon tip and electroporated at the following conditions: 1600 V, 10 ms, 3 pulses. Then, the cells were transferred to a 24-well plate and dynabeads were added at the ratio beads: cells 1 to 1. The electroporation was repeated for positive and negative controls from each donor. The transfected cells were incubated in a humidified 37 °C, 5%CO2 incubator for 48–72 h.

#### Cell activation and expansion

48–72 h after nucleofection, fresh medium with 20U/ml of IL-2 was added to the cells and 48–72 h later, the cells were stained with anti-CD27 and the efficiency of nucleofection was measured as the percentage of CD27 on CD4 T cells detected by flow cytometer. The cells were fed with fresh medium and IL-2 (20U/ml) for two weeks and then, the cells were washed, suspended in fresh medium, and seeded at the concentration of 2 × 10^5^ cells/well in a U-bottom, 96-well plate pre-coated with 10 ug/ml of anti-CD3. At 24- and 48-hours supernatant was analysed for levels of IFNg and protein extraction from cell pellet was performed.

### Purification of extracellular vesicles

Extracellular vesicles were isolated from supernatants of isolated CD4 T cells cultured with or without anti-CD3 (5 μg/ml) for 72 h using the methodology described in the paper [25].

In brief, the culture supernatants were centrifuged at 2,000 g for 20 min to remove debris and dead cells. Then the supernatants were centrifuged at 16,500 g for 45 min to isolate endosomal vesicles of 10k. The supernatants were centrifuged at 100,000 g for 2 h at 4 degrees and the pellets were resuspended in PBS and centrifuged at 100,000 g for 2 h a room temperature to isolate endosomal microvesicles of size 100k (exosomes) [26]. Cell and endosomal vesicle pellets were resuspended in a cell extraction buffer for total protein extraction as described above.

### Western blot analysis

Proteins extracted from whole cells and exosomes were separated by 4–12% SDS-PAGE (Mini protean TGX stain-free gels, Bio-Rad, UK) and transferred onto PVDF membranes previously activated in methanol for 30 s. The blots were blocked with 5% non-fat dry milk a room temperature for 1 h and incubated with the working concentration of primary antibodies overnight at 4 °C and then incubated with HRP-conjugated secondary antibodies (Invitrogen) a room temperature for 1 h. The membranes were developed with an enhanced chemiluminescence (ECL) detection kit (Pierce). Information about the primary and secondary antibodies is reported in Supplementary Table 3.

### Post-mortem CSF and brain tissues of patients with MS and controls

Snap-frozen brain and/or paraffin-embedded 4% paraformaldehyde-fixed (FFPE) tissues and matched CSF from a total of 55 cases with a diagnosis of SPMS and 17 control cases were provided by the UK Multiple Sclerosis Society Tissue Bank (Imperial College London, UK). Demographic and clinical features of MS cases were described in Supplementary Tables 4 and control cases in Supplementary Table 5. FFPE tissues from 35 out of the 55 examined post-mortem MS cases were used for neuropathological investigation. The combined CSF samples were analysed for protein levels of sCD27 and sCD21 by using the R&D system Elisa kit and analysed by Simple Plex (ProteinSimple, San Jose, CA) following the manufacturer’s instructions. In addition, the protein levels of CCL19, CXCL10, CXCL12, CXCL13, IL-6, IL-10, BAFF, and IFN-g were determined using customised immune-assay multiplex Luminex technology (Bio-Plex X200 System equipped with a magnetic workstation, Bio-Rad, Hercules, CA, USA), following the procedure previously optimized [27]. NFL proteins were measured using the ELISA assay (MyoBioSource San Diego, CA, USA) according to procedures previously optimized [27].

### Immunohistochemistry

Snap-frozen 10 μm sections were fixed with cold methanol for 5 min. The sections were then washed with PBS, treated with a solution 0.3% hydrogen peroxidase blocked with a solution of 2.5% horse serum and incubated with a working solution of primary antibody (anti-human CD3, anti-human CD27 and anti-human CD20) overnight at 4^o^C in a damp container. Antibody binding was detected by horseradish peroxidase-conjugated secondary antibody using an automated chromogenic detection system. The slides were washed in running water for 5 min, stained in haematoxylin for 1 min, washed for 5 min in water and dipped in solutions with rising percentages of ethanol 70%, 80%, 100% and 100% of Xylene. A drop of aqueous mounting solution was added on each slide that was closed with a coverslip and left to dry overnight. The images were acquired by using slide scanner Leica biosystem Aperio AT2 and the cell count was performed by ImageJ Fiji software.

### Immunofluorescence

An Immunofluorescence assay was performed on snap-frozen sections from the brain tissues of 2 patients. A blocking solution of 2.5% of horse serum in PBS was added to each section and then a working solution of a combination of primary antibodies (one combination of antibodies included mouse anti-human CD4, goat anti-human CD27, rabbit anti-human CD8, and a second combination included mouse anti-human CD3, goat anti-human CD27 and rabbit anti-human CD20) was added overnight in a humid container at 4 °C. After three washes with PBS, a solution of anti-rabbit biotin antibody in 2.5% horse serum was added for 1 h. The sections were washed three times and a working solution of secondary fluorescence antibodies (donkey anti-mouse 555, donkey anti-goat 488 and donkey streptavidin-647) was added for 2 h. Subsequently, the sections were washed three times with PBS and a DAPI solution (Roche Diagnostics GmbH, Germany) was added for one minute. After washes, the slides were mounted with antifade medium (Vectashield, Vector laboratories), coverslipped and dried for almost 2 h in a dark room. The images were obtained by using a Leica TCS SP8 confocal microscope. Antibodies used in immunohistochemistry and immunofluorescence are given in Supplementary Table 6.

### Neuropathological characterization of brain tissues

A semiquantitative scoring system was developed in a subgroup of 35 MS cases to have a standardised measure of the degree of demyelination and inflammation in the different compartments examined (meninges, perivascular spaces, choroid plexus). The presence and extent of demyelination and the activity of MS cases were assessed by combining immunohistochemical detection of myelin oligodendrocyte glycoprotein (MOG) and major histocompatibility complex (MHC) class II combined with Luxol fast blue (LFB) staining on serial sections, as previously described [28, 29]. The percentage area of demyelinated white matter (WM) and grey matter (GM) was measured using Qpath [30]. The entire GM and WM fraction for each MOG immunostaining was traced and measured, then the area of individual GM and WM lesions was measured. Finally, the mean GM and WM lesion area was reported per section and MS case as the percentage of total demyelinated GM or WM, respectively. In addition to the detection of lymphoid-like structures in the meninges, a score of the degree of inflammation in the different compartments (choroid plexus and perivascular infiltrates) was performed on a semi-quantitative basis, rating the degree of inflammation in three different levels of intensity by examining the haematoxylin-eosin staining of all the examined blocks. According to previously published procedures [31], score 0 corresponds to absent/scarce (0–5 cells), 1 corresponds to moderate (6–25 cells), 2 corresponds to high (25–50 cells), and 3 (> 50 cells) corresponds to elevated inflammatory infiltrate. All semi-quantitative scoring was performed on images acquired at 20x objective (field area 556.5 μm x 312,9 μm equal to 174128,85 μm²).

### R analysis

PCA and HCA were made with R Studio version 4.0.2, Factoextra (10.7) and factoMiner packages, missMDA package for missing values., Corrplot and ggplot2 packages for hierarchical clustering, To establish whether sCD27 is a predicting factor for a specific neuropathological variable, we adopted the Random Forest Recursive Feature Elimination method (RF-RFE) [32]. In detail, a 10-iteration 5-fold cross-validation protocol was adopted, and performances were measured using the Mean Squared Error. The analyses were performed with MATLAB version R2022b.

### Statistical analysis

Statistical analyses were performed using Prism version 9.2 (GraphPad Software Inc.) and MATLAB. Differences between groups were compared using the Mann-Whitney test, 2way Anova multiple comparisons, Ratio paired t-test, one sample t-test, Kruskal-Wallis test, and Dunn test. Correlations were assessed using the Spearman rank correlation. Flow cytometry analyses were performed using Flow-jo software version 10.6.1 (FlowJo LLC).

## Results

### CSF sCD27 correlates with inflammatory lymphocytes in MS patients

**Fig. 1:**
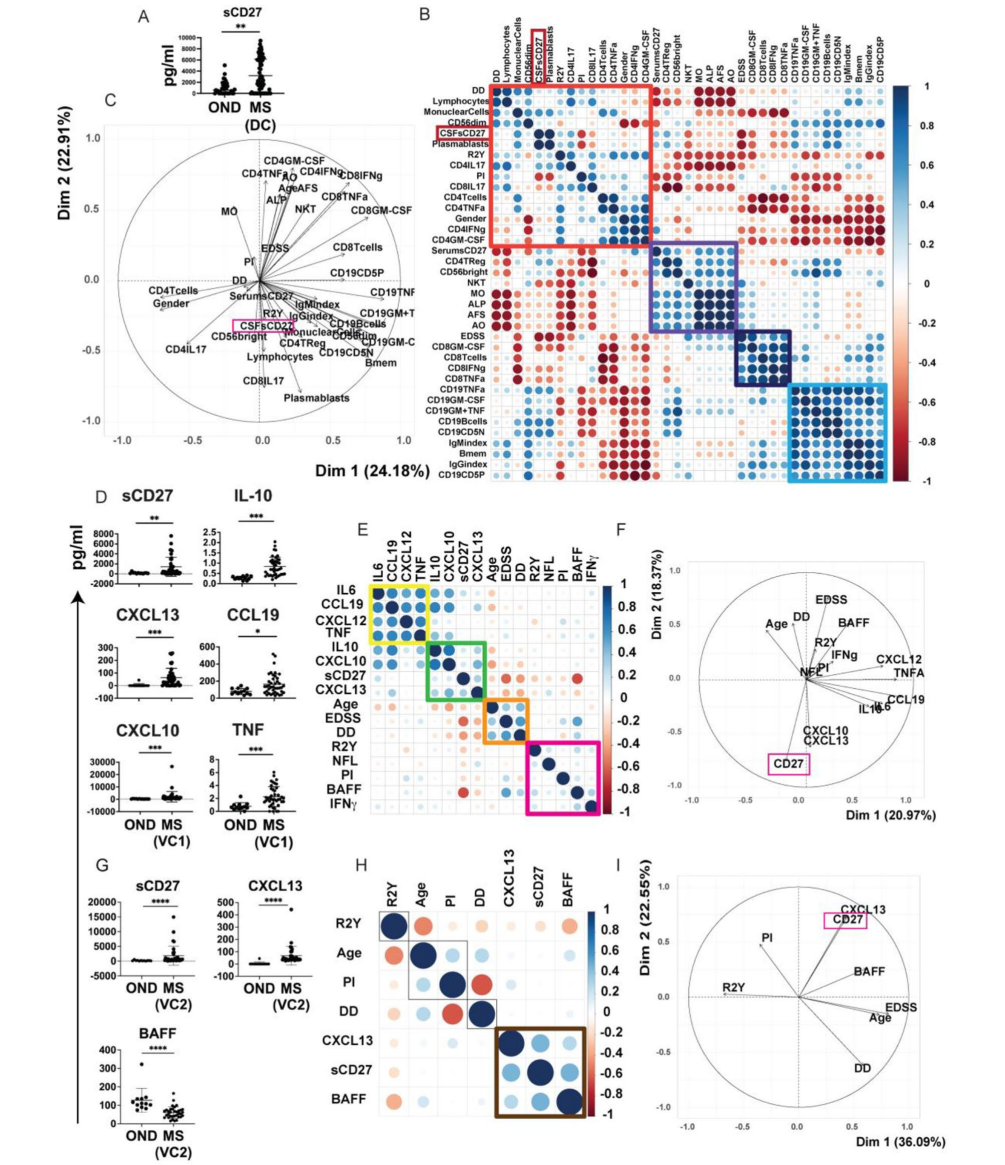
CSF sCD27 correlates with inflammatory lymphocytes in MS patients. (**A**) Levels of sCD27 in the CSF of patients with MS discovery cohort (DC) compared to other neurological disease (OND) as measured by ELISA. Plots display individual levels (dots) and mean (horizontal line) of 65 MS and 35 OND patients, statistically compared by Mann-Whitney test. (**B**) HCA and (**C**) PCA of CSF analytes and features of MS DC. (**D**) Plots compare the CSF levels of sCD27, CXCL13, CXCL10, CCL19, TNF and IL-10 in MS VC1 and patients OND as measured by ELISA. Plots display results from 41 MS validation cohort (VC)1 and 12 OND, statistically compared by Mann-Whitney test. (**E**) HCA and (**F**) PCA of CSF analytes and features of MS VC1. (**G**) Plots compare CSF levels of sCD27, BAFF and CXCL13 in MS VC2 and OND measured by ELISA. Plots display results from 30 patients with MS and 12 OND. Statistical analyses were performed by using Mann-Whitney test. (**H**) HCA and (**I**) PCA of CSF analytes and features of MS VC2

We first measured the levels of sCD27 in the CSF alongside several other variables in a discovery cohort (DC) of 65 patients with RR-MS (MS DC). In the MS DC, CSF sCD27 was significantly higher (**p < 0.001) than in a group of 35 patients with OND as shown in Fig. 1A. MS variables from this discovery cohort including clinical data, percentage of CSF mononuclear cells, levels of CSF soluble factors and levels of sCD27 in serum and CSF were examined in a heatmap correlation matrix with hierarchical clustering (HCA) (Fig. 1B). The heatmap showed 4 principal clusters: Cluster 1 (red), inflammatory interferon (IFN), Tumour necrosis factor (TNF), interleukin (IL)-17 and granulocyte-macrophage colony-stimulating factor (GM-CSF)-producing CD4 T cells, IL-17-producing CD8 T cells, plasma blasts, levels of CSFs CD27, disease duration (DD), gender, progression index (PI) and the number of relapses within 2 years (R2Y); Cluster2 (violet), sCD27 levels in serum, frequencies in CSF of CD4Treg, Natural Killer (NK)T cells, CD56^Bright^ NK cells, monocytes, age at onset (AO), age at the first symptom (AFS) and age at lumbar puncture (ALP); Cluster 3 (black), inflammatory GM-CSF, IFN-γ, TNF-producing CD8 T cells and expanded disability status scale (EDSS) score; and Cluster 4 (blue), CD19 B cells, GM-CSF, TNF-producing CD19 B cells, immunoglobulin (Ig)M and IgG index, memory B cells and CD19 CD5+/- B cells. Levels of CSF sCD27 were positively correlated with the percentage of mononuclear cells, plasma cells, inflammatory IL-17, IFNg and TNF-producing CD4 T cells, R2Y and negatively correlated with IL-17-producing CD8 T cells and PI (Fig. 1B). The principal component analysis (PCA) showed that sCD27 constituted one of the key markers for patient clustering with IL-17-producing CD4 and CD8 T cells, CD4Treg, CD56^Bright^ NK cells and plasma blasts in the CSF. PCA showed also that inflammatory CD19 B cells and inflammatory CD8 T cells generated different patient clustering not associated with levels of sCD27 (Fig. 1C).

Considering the association of sCD27 with inflammatory T lymphocytes in the CSF, we carried out a detailed analysis of the CSF to investigate the potential correlation of other inflammatory factors with sCD27 for patient clustering. CSF samples from a first validation cohort (VC) of RR-MS patients (MS VC1) were tested for several pro-inflammatory and anti-inflammatory cytokines (IFN-γ, TNF, IL-10, and IL-6), chemokines (CXC motif chemokine ligand 12 (CXCL12), CXCL13, CXCL10, C-C motif ligand 19 (CCL19), neurofilament light chain (NF-L) and sCD27. Amongst them, sCD27 (**p < 0.006), CXCL13 (***p < 0.001), CXCL10 (***p < 0.001), CCL19 (*p < 0.014), TNF (***p < 0.001) and IL-10 (***p < 0.001) were significantly increased in MS VC1 compared to OND (Fig. 1D). Furthermore, the HCA (Fig. 1E) showed 4 principal clusters: Cluster A (yellow), IL-6, CCL19, CXCL12. and TNF; Cluster B (green), IL-10, CXCL13, CXCL12; Cluster C (orange), AO, DD and EDSS score; Cluster D (purple), R2Y, PI, IFN-γ, NFL and B-cell activating factor (BAFF). Levels of sCD27 correlated positively with CXCL13. and in PCA sCD27 is one of the key markers for patients clustering with CXCL13, and CXCL10. (Fig. 1F). We then examined a subset of factors of interest in a second, distinct validation cohort of CSF from RRMS patients (MS VC2). The levels of sCD27, CXCL13 and BAFF were significantly different in MS VC2 compared to OND. sCD27 and CXCL13 were confirmed as being higher in MS (***p < 0.001), while BAFF was significantly lower (****p < 0.0001) (Fig. 1G). In the HCA, sCD27 clustered with CXCL13 and BAFF (Fig. 1H, cluster brown). PCA confirmed the cluster of sCD27 and CXCL13 in RRMS (Fig. 1I). Taken together, the data suggest that levels of sCD27 are correlated with inflammatory CD4 T cells and are a relevant marker for patient clustering with IL-17-producing CD4 and CD8 T cells and CXCL13. The different correlation between the levels of sCD27 and BAFF in the two validation cohorts could be associated with intrathecal B cell activity. The clustering of sCD27 and CXCL13 was confirmed in both validation cohorts.

### CD27 cleavage is triggered by TCR activation

To assess the producer of sCD27, sCD27 was investigated in B and T cells.

**Fig. 2:**
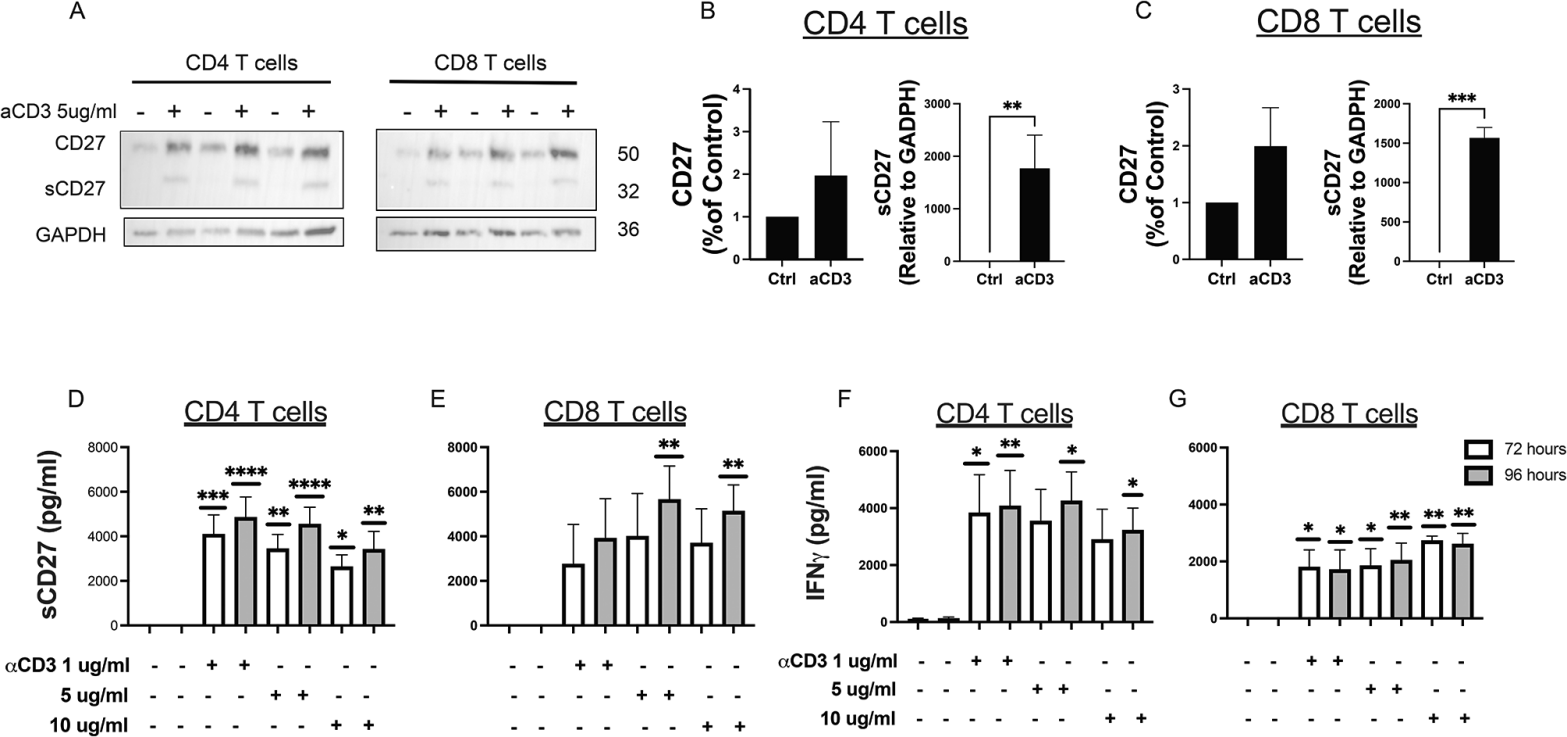
CD27 cleavage is triggered by TCR activation. (**A**) Western blot analysis of CD27 in whole cell lysate from CD4 and CD8 T cells in resting and stimulated with 5μg/ml of anti-CD3 Ab at 72 h. Quantification of CD27 as a percentage of control and sCD27 as a value relative to normalization with GADPH in CD4 (**B**) and CD8 (**C**) T cells by western blotting. Data were reported as mean ± SD of 3 independent experiments. Statistical analyses were performed using One sample t and Wilcoxon test. NS = not significant, *p < 0.05, **p < 0.01. Levels of sCD27 and IFNγ- released from CD4 (**D**, **F**) and CD8 (**E**, **G**) T cells activated with concentrations of anti-CD3 including 1, 5 and 10 ug/ml at 72h and 96h as measured by ELISA. Statistical analyses were performed using Tukey’s multiple comparison tests two-way ANOVA (**D**, **F**) and Tukey’s multiple comparison test for mixed-effects analysis (**E**, **G**). Data represent mean ± SD of 6 (**D**, **F**) and 4 independent experiments (**E**, **G**).*p < 0.05,**p < 0.01,****p < 0.0001.

CD4 and CD8 T cells were activated with 5ug/ml of anti-CD3 that cross-links T cell receptor (TCR), for 72 h before cell lysis for western blot analysis. The immunoblot for CD27 showed two bands related to the cleaved form at 32 kDa (sCD27) and not cleaved form at 50 kDa (Fig. 2A). The normalized levels of the two forms of CD27 showed an intracellular production of CD27 cleavage upon TCR activation in both CD4 and CD8 T cells as reported in Fig. 2B and C.

Protein analysis was performed in B cells isolated from 4 healthy donors and stimulated with goat anti-human IgMIgG, (H + L) functional grade that promotes B cell survival and activation, CD40L (1ug/ml) that induces the B cell activation through the CD40 receptor, CpG ODN 2006 (2.5ug/ml), a synthetic agonist of Toll-like receptor (TLRs) 9 and R848 (1ug/ml,) a synthetic agonist of TLR 7 and 8 [33], for 48 h, revealed the presence of bands for sCD27 and CD27 in western blot analysis (supplementary Fig. 1A). Levels of CD27 and sCD27 do not change in the stimuli compared to the control (supplementary Fig. 1B and C).

Protein levels of sCD27 and IFNγ- were measured in supernatants collected at 72h and 96h from cultures of CD4 and CD8 T cells activated with anti-CD3. Significant levels of sCD27 were observed in the supernatants of activated CD4 T cells stimulated with 1, 5 and 10 µg/ml of anti-CD3 Ab at 72 and 96 h (Fig. 2D) and in activated CD8 T cells stimulated with 5 and 10 µg/ml of anti-CD3 Ab at 96 h (Fig. 2E) compared to untreated cells. Significant levels of IFN-γ were observed in CD4 T cells activated with 1 µg/ml of anti-CD3 at 72h and 96h and 5, 10 µg/ml of anti-CD3 at 96 h (Fig. 2F) and in CD8 T cells activated with 1, 5 and 10 µg/ml of anti-CD3 at 72h and at 96h (Fig. 2G) compared to untreated cells.

Protein levels of sCD27 and TNF were investigated in the supernatants of B cells stimulated with anti-human IgM/IgG, (H + L) functional grade, CD40 ligand (CD40L), CpG and R848 for 12, 24 and 48 h. In this experiment, B cells released significant levels of sCD27 upon stimulation with IgM/IgG, (H + L) functional grade at 48h (supplementary Fig. 1D). Significant release of TNF was observed in B cells stimulated with R848 at 12, 24 and 48h (supplementary Fig. 1E). Although we observed a significant release of sCD27 from B cells activated by cross-linking of BCR in the ELISA analysis, the results have not been supported by the protein analysis with western blotting where the levels of sCD27 produced from B cells are not significant in activated B cells compared to the control. In addition, activated B cells released sCD27 only up to 200 pg/ml, while activated T cells released sCD27 in a range of 2000–6000 pg/ml, stimulating the same number of cells (2 × 10^5^ cells/well). Based on these results we conclude that activated T cells, compared to B cells, release more sCD27 and do so by a mechanism involving TCR-mediated activation.

### TCR activation releases extracellular vesicles carrying CD27

**Fig. 3:**
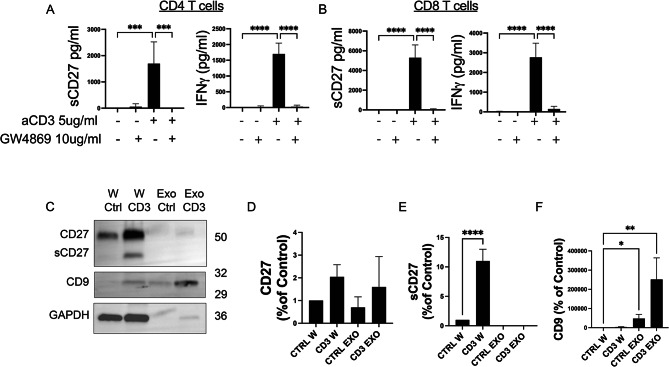
TCR activation releases extracellular vesicles carrying CD27. Levels of sCD27 and IFN-γ- released from CD4 (**A**) and CD8 (**B**) T cells in resting or stimulated with anti-CD3 when they are pretreated or not with GW4868 for 72 h as measured by Elisa. Data represent mean ± SD of 4 independent experiments. Statistical analyses were performed using ordinary one-way ANOVA. NS = not significant, p***<0.001, p****<0.0001. (**C**) Immunoblot for CD27 and CD9 in the whole cell lysate (W Ctrl and W CD3) and purified exosomes (Exo Ctrl and Exo CD3) from purified CD4 T cells in resting and stimulated with anti-CD3.The same amount of protein from the exosome and whole cell lysate were loaded. Quantification of CD27 (**D**), sCD27 (E) and CD9 (F) as a percentage of control in the whole cell lysate and purified exosomes in resting and stimulated with anti-CD3 Ab by western blotting. The experiments were repeated 3 times independently. Data represent mean ± SD. Statistical CD27 analyses were performed using one-Anova way for multiple comparisons. ****p < 0.0001

We hypothesized that sCD27 is released through an exocytosis mechanism triggered by TCR activation. To test this hypothesis, CD4 and CD8 T cells were pre-treated with GW4869 (10 µg/ml) which inhibits nSMase, and then stimulated with 5 µg/ml of anti-CD3 Ab for 72 h. Levels of sCD27 and IFNg were significantly reduced in CD4 (Fig. 3A) and CD8 (Fig. 3B). Furthermore, to investigate whether sCD27 is present in extracellular vesicles generated after TCR engagement, CD4 T cells were activated with 5 µg/ml of anti-CD3 for 72h and extracellular vesicles were isolated from the medium. Proteins were then extracted from exosomes and whole cell lysates and levels of CD27, CD9 (exosome marker), and GAPDH were assessed by western blotting (Fig. 3C). After normalization, the data showed that levels of CD27 were present on protein extracted from exosomes and whole cell lysate of CD4 T cells and that levels of this protein did not change in T cells and exosomes upon activation (Fig. 3D). In addition, levels of sCD27 were only observed in protein extracted from whole cell lysate of CD4 T cells upon activation (Fig. 3E). Those results demonstrated that sCD27 production and release is mediated by TCR activation and is not released in microvesicles.

### Knocking out CD27 reduces IFN-γ-production in CD4 T cells

**Fig. 4:**
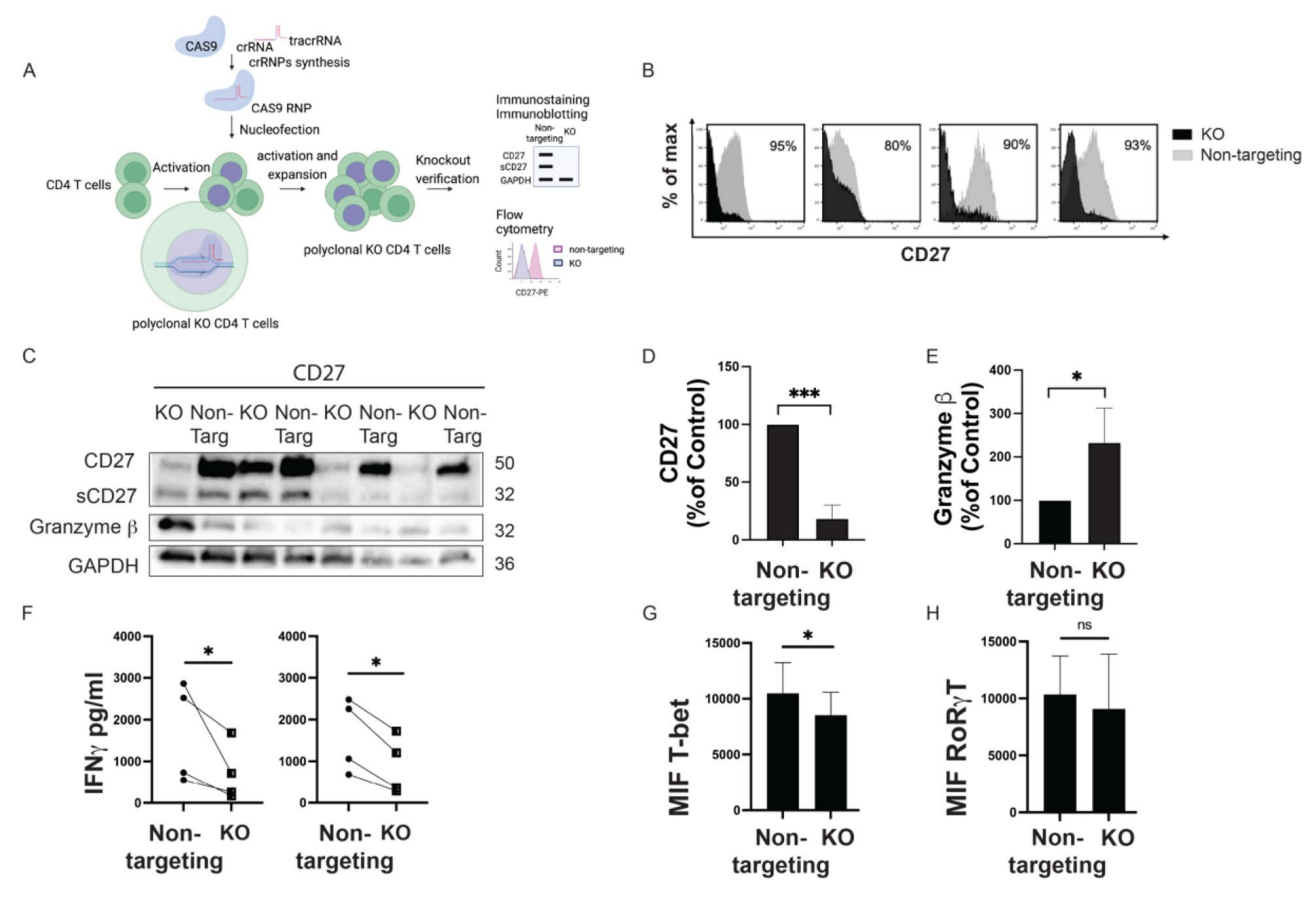
CD27 editing reduces IFNγ-producing CD4 T cells. (**A**) The CD27 gene was efficiently “knocked out” in CD4 T cells by using CRISPR-Cas9-single-guide RNA ribonucleoproteins (Cas9-RNP). CRISPR-cas9 ribonucleoproteins (crRNPs) were synthesized in vitro and delivered to activated CD4 T cells by nucleofection for editing. The expression of CD27 on the surface of CD4 T cells was investigated by flow cytometry and immunoblotting. (**B**) Flow cytometry histograms showing CD27 levels on the cell surface of purified CD4 T cells treated with non-targeting crRNPs (non-targeting, in grey) or CD27-targeting crRNP (KO, in dark) in 4 independent experiments. (**C**) Immunoblots for CD27 and granzyme B in the whole cell lysate of CD4 KO and CD4 non-targeting T cells. Quantification of CD27 (**D**) and (**E**) granzyme B as a percentage of control by western blotting. Data represent mean ± SD of 4 independent biological experiments. Statistical analyses were performed using one-tailed t and Wilcoxon test. *p < 0.05, ***p < 0.001. (**F**) Levels of sCD27 (pg/ml) from stimulated CD4 KO and CD4 non-targeting T cells were compared at 24 h and 48 h as measured by ELISA. Statistical analyses were performed using ratio paired t-test. *p < 0.05. Histograms reporting intracellular levels of T-bet (**G**) and RoR-γT (**H**) in CD4 KO and CD4 non-targeting T cells as measured by flow cytometry. T-bet and RoR-γT were reported as mean fluorescence intensity (MFI). Data represent mean ± SD. Statistical analyses were performed using paired t-tests of 4 independent experiments. NS not significant, *p < 0.05

The analysis of CSF in the discovery cohort demonstrated a correlation between levels of sCD27 and inflammatory CD4 T cells and for this reason, we have focused on CD4 T cells. As CD27 changes after activation on CD4 T cells, to investigate whether sCD27 contributes to the differentiation of IFN-γ-producing cells, the CD27 gene was efficiently “knocked out” in CD4 T cells by using CRISPR-Cas9 RNPs (Fig. 4A). The gene editing caused the ablation of CD27 on 80–90% of CD4 + T cells knocked down for CD27 (KO) compared with control (non-targeting CD4 T cells) (Fig. 4B). Furthermore, proteins extracted from KO and non-targeting CD4 + T cells were analyzed by western blotting (Fig. 4C) and the analysis demonstrated a significant decrease of CD27 (Fig. 4D) and significant increase of levels of granzyme b (Fig. 4E) in KO compared to non-targeting CD4 + T cells.

Reduced IFN-γ production was observed in supernatants of KO compared with non-targeting CD4 T cells collected after 24 and 48 h of TCR activation (10 µg/ml anti-CD3) (Fig. 4F). Flow cytometric analysis of levels of intracellular transcriptional factors showed a significant reduction of levels of transcriptional factor T-bet in KO compared to non-targeting CD4 + T cells (Fig. 4G) and similar levels of the retinoid orphan receptor (ROR)-gT in KO and non-targetingCD4 + T cells (Fig. 4E). Our experiment demonstrated that sCD27 may be relevant in the differentiation of Th1 CD4 T cells and its effect may be mediated via regulation of T-bet expression.

### Levels of sCD27 in CSF are correlated with CD27 + T cell density in SPMS post-mortem CNS tissues

**Fig. 5:**
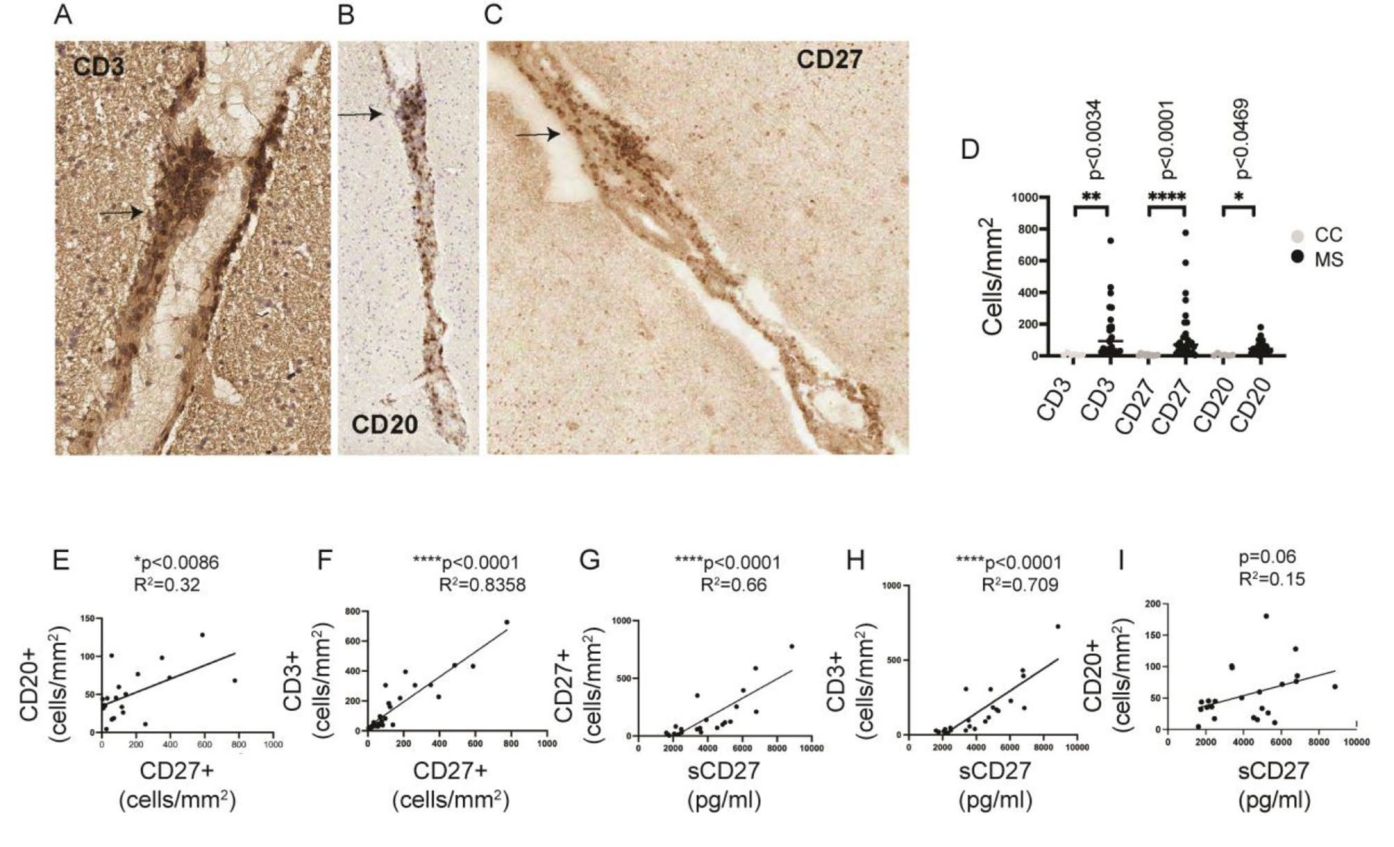
Levels of CSF sCD27 were significantly correlated to CD3 and CD27 cell density in matched post-mortem brain tissues of patients with SP-MS. Representation of immunohistochemistry staining of CD3+(**A**), CD20+ (**B**) and CD27+ (**C**) in perivascular cuffs on sections of snap-frozen post-mortem brain tissues from patients with MS. The arrows indicate the positive cells for CD3, CD20 and CD27. (**D**) Scatter plot reporting the cell density of CD3, CD27 and CD20 cells detected in the perivascular cuffs and meninges in 6 control (grey dots) and 27 MS cases (black dots). Data represent mean ± SD of 6 control cases, 24 MS cases for aCD3, 27 MS cases for CD27, and 23 MS cases for CD20. Statistical analyses were performed by using one-way ANOVA, Kruskal Wallis test with Dunn’s multiple comparison test. *p < 0.05, **p < 0.01, ***p < 0.001. Simple linear regression between CD27 cell density and CD20 cell density (**E**), and between CD27 cell density and CD3 cell density (**F**) detected in perivascular cuffs and meninges. *P* and r^2^ squared are shown in the legends. Simple linear correlation between levels of CSF sCD27 (pg/ml) and CD27 cell density (**G**), CD3 cell density (**H**) and CD20 cell density (**I**). *P* and r^2^ are reported in the plot’s legends

Our results indicated that sCD27 in the CSF of patients with MS could promote activation and differentiation of inflammatory T cells, a likely relevant effect in the disease pathophysiology. To examine this hypothesis in CNS tissue, we have analyzed the percentage of CD27 + T and B cell infiltrates and the neuropathology of post-mortem brain tissues from patients with MS and tested the associations with sCD27 in the CSF. Levels of sCD27 were analyzed in the CSFs isolated post-mortem from 27 individuals with SPMS and 9 controls. In parallel, the cell density of CD3, CD20 and CD27 (Fig. 5A-C) cells was evaluated in the immune infiltrates of perivascular cuffs in the WM and meninges of matched snap-frozen brain tissues. Significantly increased number of CD3+ (**p < 0.0034), CD27+ (****p < 0.0001) and CD20 + cells (*p < 0.0469) were detected in perivascular and meningeal infiltrates of MS cases compared to controls (Fig. 5D). A significant correlation was observed between CD27 and CD20 positive cells (Fig. 5E *p < 0.0086), as well as CD3-expressing cells (Fig. 5F ****P < 0.0001). The levels of CSF sCD27 correlated significantly to the number of CD27+ (Fig. 5G, ****p < 0.0001) and CD3 + T cells (Fig. 5H, ****p < 0.0001) (cells/mm2), but not with CD20 + cells (Fig. 5I, *p* = 0.06) counted in the same MS cases.

**Fig. 6:**
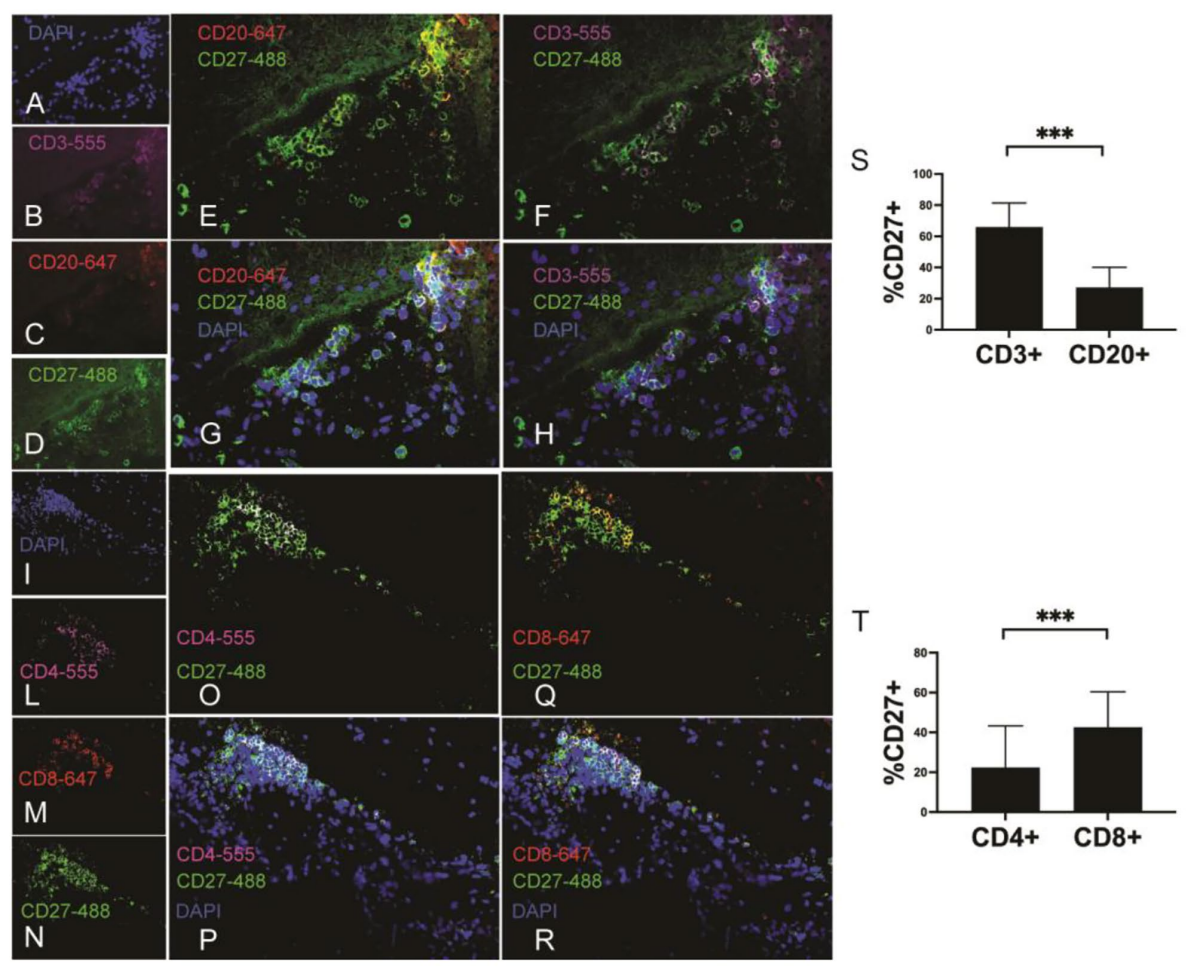
CD27 expression on T cells in CNS from MS brain. Representation of immunofluorescence on sections of snap-frozen post-mortem brain tissue performed with 4 colour staining. Single labelling DAPI (**A**, blue), aCD3 Alexa Fluor 555 (**B**, purple), CD20 Alexa Fluor 647 (**C**, red) and CD27 Alexa Fluor 488 (**D**, green). Colocalization of CD27 on CD20 B cells (**E**-**G**) and CD3 T cells (**F**-**H**). Single staining DAPI (**I**, blue), CD4 Alexa fluor 555 (**L**, purple), CD8 Alexa fluor 647 (**M**, red) and CD27Alexa fluor 488 (**N**, green). Colocalization of CD27 on CD4 T cells (**O-P**) and CD8 T (**Q-R**). (**S**) histograms report the percentage of CD27 + cells on CD3 and CD20 cells and (**T**) on CD4 and CD8 T cells detected in perivascular cuffs and meninges. Data represent mean ± SD. Statistical analysis was performed by using the Mann-Whitney test. ***p < 0.001

To define the immune profile of CD27 + cells, 4-colour immunofluorescence was performed on post-mortem brain tissues from two patients with MS. CD27 was detected on B and T cells (Fig. 6A-H) and CD4 and CD8 T cells (Fig. 6I-R). More CD3 T cells co-express CD27 than CD20 B cells (Fig. 6S, ***p < 0.001) and more CD8 than CD4 T cells (Fig. 6T, ***p < 0.001).

### CSF sCD27 levels are increased in post-mortem cases with active MS lesions

**Fig. 7:**
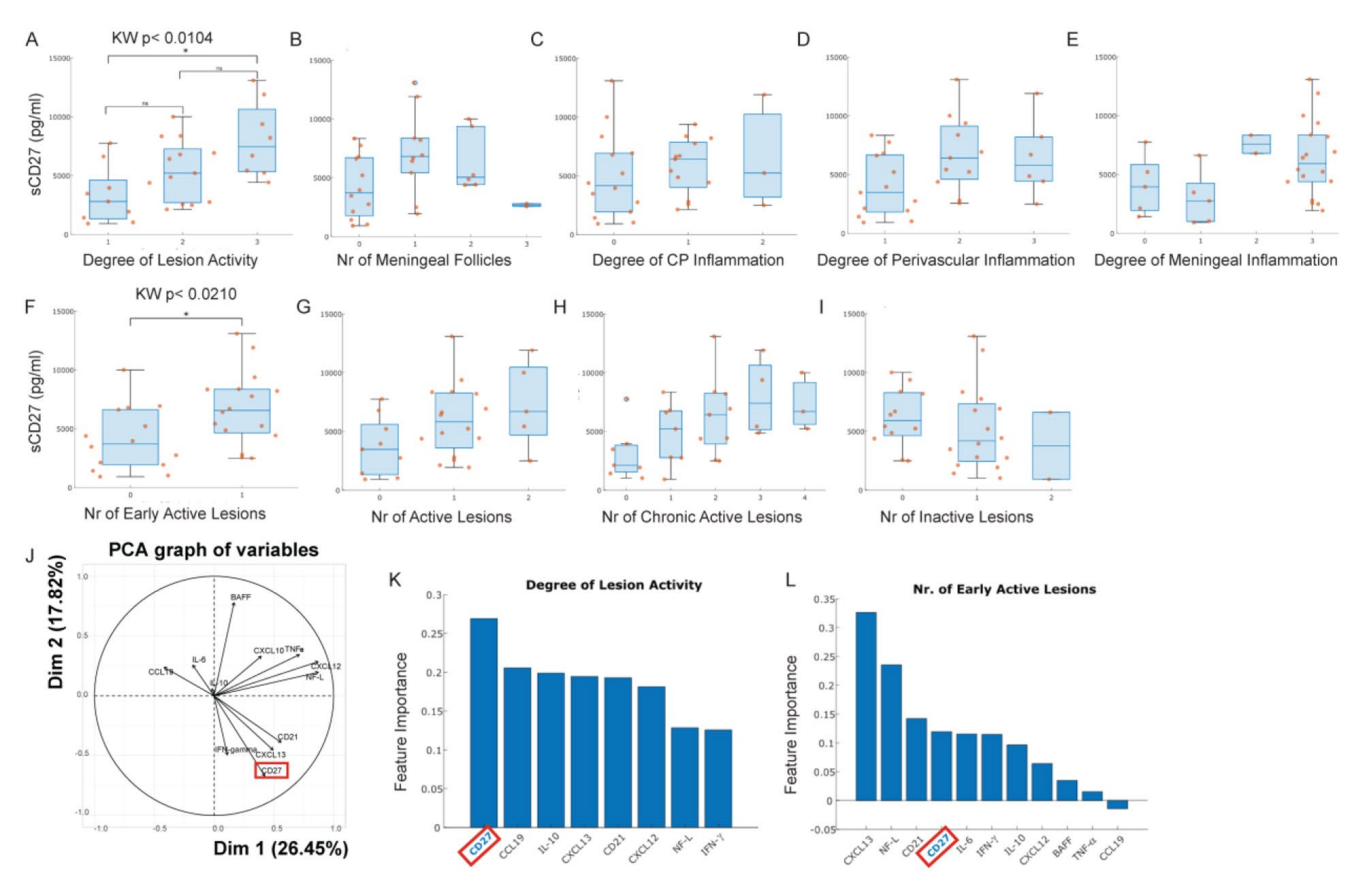
sCD27 in post-mortem MS cases reflects the degree of lesion activity. Histograms reported levels of sCD27 in different groups defined by degree of lesion activity (**A**), number of meningeal follicles (**B**), degree of CP (choroid plexus) inflammation (**C)**, degree of perivascular inflammation (**D**), degree of meningeal inflammation (**E**), number of early active lesions (**F**), number of active lesions (**G**), number of chronic active lesions (**H**), number of inactive lesions (**I**). The data represent mean ± SD of 30 post-mortem MS cases. Statistical analyses were performed by using one-way non-parametric ANOVA and Mann-Whitney U test (**F**). Principal component analysis of cytokines detected in the matched CSF of 30 post-mortem MS cases (**J**). Histograms report the features stratified for importance for degree of lesion activity (**K**) and number of early active lesions (**I**) obtained from Random Forest (RF)-recursive feature elimination (RFE) algorithm

To examine potential neuropathological associations, the levels of sCD27 were compared across different groups, which were obtained by stratifying 30 out of 55 of the examined MS cases according to each neuropathological variable. sCD27 was found to be distributed in a significantly different way according to the degree of lesion activity, graded as defined in the Methods (Fig. 7A Kruskal-Wallis *p*-value 0.0104); in particular, sCD27 was significantly higher (Dunn test *p*-value 0.0077) in the group of subjects with the highest inflammation degree of lesion activity (degree equal to 3) than in the group with lower one (inflammation degree equal to 1). Furthermore, the sCD27 was found to be significantly increased in subjects with early active lesions (Fig. 7F Mann-Whitney U test *p*-value 0.0210), while the seven remaining neuropathological features did not have any influence on the levels of sCD27 (Fig. 7B-E, G-I). The principal component analysis (PCA, Fig. 7J) of 12 CSF markers, including CXCL10, CCL19, TNF, BAFF, CXCL12, soluble CD21, CXCL13, IL-10, NFL, IL-6, IFN-g and sCD27 showed sCD27 clustering together with IFN, and the CXCL13 and the soluble complement receptor type 2 (CD21) (Fig. 7J).

### sCD27 is a predictive factor of extensive active inflammatory lesions in postmortem MS cases

For each neuropathological feature, 12 CSF markers measured on 30 post-mortem subjects were imputed to the RF- RFE algorithm. Among the 9 sets of markers returned by the RF-RFE, 2 of them included sCD27. In detail, sCD27 is the marker with the highest importance in predicting the degree of lesion activity, in a signature made up of 8 CSF markers (Fig. 7K). sCD27 is also one of the markers making up the signature of the number of early active lesions (Fig. 7L): it is the 4th in terms of importance, which is comparable to other molecules for this feature.

## Discussion

In this study, we demonstrate that sCD27 is increased in CSF from patients with MS, is correlated with inflammatory T cells and is a key marker for the clustering of patients with intrathecal lymphocyte infiltrates and CXCL13. Our in vitro studies demonstrate that sCD27 is produced intracellularly in T cells by cross-linking of TCR and released by a secretory mechanism mediated by sphingomyelinase. CD27 is released in soluble form and micro-vesicles and may promote the differentiation of inflammatory effector, IFNγ-producing T cells by modulating T-bet expression. Results from our analysis of matched CSF and brain tissues from post-mortem MS cases demonstrate that sCD27 in the CSF of patients with MS is a marker of inflammatory lesions generated by intrathecal T-cell activity.

We find a different pattern of BAFF in the CSF of MS patients associated with patient clustering. Analysis of CSF showed that lower levels of BAFF in the CSF are associated with higher production of sCD27 and in those MS patients we observe a clustering of CSF sCD27, BAFF and CXCL13 supporting a contribution of B cells to T cell activation and/or the involvement of sCD27 to B cell activation [34–36]. In MS, BAFF is produced in the CNS by astrocytes [34] located in the perivascular area and parenchyma of chronic and acute lesions and promotes the persistence and clonal expansion of BAFF-R-expressing B cells [35] and T cell activation [36]. A clustering of CSF sCD27, CXCL13 and CXCL10 is observed in MS cases with normal levels of BAFF. CXCL10 is a chemokine which is induced by IFN-g and is crucial for T cell lymphocyte trafficking to inflamed tissue [37, 38]. In previous reports, higher CSF CXCL10 in patients with MS was associated with an increased leukocyte number in the CSF [39] and demyelination [40]. In actively demyelinating lesions examined in post-mortem specimens from MS cases, CXCL10 is produced by macrophages within the lesion and by reactive astrocytes present in the surrounding parenchyma [41]. We found a clustering of sCD27, CXCL13 and soluble CD21 in the CSF of progressive post-mortem cases, suggesting a potential link between sCD27 and elevated intrathecal B cell activity in the progressive phase, possibly localised in meningeal lymphoid-like structures. Levels of CXCL13 were demonstrated to be more associated with peripheral lymphocyte trafficking in the CNS [42] and Th1-mediated inflammatory process in the CNS [9] than with production from the CNS-resident meningeal and glial cells [43]. Levels of CXCL13 and sCD27 in the CSF of patients with active RR-MS undergoing autologous haematopoietic stem cell transplantation (AHSCT) and showing remission of disease activity over the 5-year follow-up after AHSCT decreased to levels of controls for all the period study suggesting that the long-lasting remission of disease activity in those patients can be associated with reducing the trafficking of lymphocytes in the CNS [44].

We demonstrate with in vitro experiments that T cells are the main lymphocyte producers of sCD27. Activated T cells produce intracellularly sCD27 that is released in the range of 2000–6000 pg/ml, whereas the same number of activated B cells only released up to 200 pg/ml sCD27. Moreover, our in vitro experiments with CD27 knockout T cells show a significant reduction of transcription factor T-bet, which is the master regulator of T helper 1 (Th1) differentiation and T-cell homing to inflammatory sites. CD27 knockout CD4 T cells show an increased production of granzyme b and reduction of IFNg release. Previous reports demonstrated that sCD27 is released by metalloproteinase. We also demonstrate that the inhibition of nSMase that regulates exocytosis significantly diminished the release of IFN-γ- and sCD27. In our experiments, protein analysis of extracellular vesicles isolated from activated T cells shows the presence only of a membrane form of CD27. nSMase has been demonstrated to be activated in response to inflammatory cytokines such as TNF and IL-1b, CD95, CD40, oxidative stress [45] and pathogens [46] and regulates different cellular processes including apoptosis, cell cycle, exosome release and activity of immune cells [47]. By examining matched CSF and tissues in post-mortem cases, we identify a correlation between sCD27 and CD27 + T cells detected in inflammatory infiltrates in perivascular areas and leptomeninges. Stratification of cases according to the degree of lesion activity and type of lesions reveals a significant association of levels of sCD27 with both the presence of high degree lesion activity and of early lesions in the WM. These results suggest that levels of sCD27 are associated with lymphocyte infiltration and T-cell-associated disease activity in the CNS of patients with MS. A limitation of this observation is that since a longitudinal neuropathological evaluation is not possible, we cannot establish when these interactions develop during the disease process. One further limitation of our study includes a relatively small number of subjects, yet they were sufficient to identify several statistically robust associations that confirm and expand current knowledge.

We conclude that sCD27, although associated with both T and B cells as well as plasma cell/blast numbers and related factors’ levels in CSF here and in previous studies, is primarily produced by T cells, is clinically and mechanistically associated with pro-inflammatory cytokine-mediated CNS injury and could be further developed as a surrogate biomarker of disease activity, potentially informative also in the monitoring of anti-inflammatory treatment effects.

### Electronic supplementary material

Below is the link to the electronic supplementary material.

Supplementary Material 1

## Data Availability

The data that supports the findings of this study are available on request from the corresponding authors.

## References

[1] Reich DS, Lucchinetti CF, Calabresi PA (2018). Multiple sclerosis. N Engl J Med.

[2] Lassmann H, Bruck W, Lucchinetti CF (2007). The immunopathology of multiple sclerosis: an overview. Brain Pathol.

[3] Kabat EA, Moore DH, Landow H (1942). An Electrophoretic Study of the Protein Components in Cerebrospinal Fluid and their relationship to the serum proteins. J Clin Invest.

[4] Villar LM, Masterman T, Casanova B, Gomez-Rial J, Espino M, Sadaba MC, Gonzalez-Porque P, Coret F, Alvarez-Cermeno JC (2009). CSF oligoclonal band patterns reveal disease heterogeneity in multiple sclerosis. J Neuroimmunol.

[5] Meinl E, Krumbholz M, Hohlfeld R (2006). B lineage cells in the inflammatory central nervous system environment: migration, maintenance, local antibody production, and therapeutic modulation. Ann Neurol.

[6] Jelcic I, Al Nimer F, Wang J, Lentsch V, Planas R, Jelcic I, Madjovski A, Ruhrmann S, Faigle W, Frauenknecht K (2018). Memory B cells activate Brain-Homing, autoreactive CD4(+) T cells in multiple sclerosis. Cell.

[7] Genetics IMS, Wellcome Trust Case Control C, Sawcer C, Hellenthal S, Pirinen G, Spencer M, Patsopoulos CC, Moutsianas NA, Dilthey L, Su A (2011). Genetic risk and a primary role for cell-mediated immune mechanisms in multiple sclerosis. Nature.

[8] Arneth B (2021). Contributions of T cells in multiple sclerosis: what do we currently know?. J Neurol.

[9] Comabella M, Montalban X (2014). Body fluid biomarkers in multiple sclerosis. Lancet Neurol.

[10] Housley WJ, Pitt D, Hafler DA (2015). Biomarkers in multiple sclerosis. Clin Immunol.

[11] Hintzen RQ, Paty D, Oger J (1999). Cerebrospinal fluid concentrations of soluble CD27 in HTLV-I associated myelopathy and multiple sclerosis. J Neurol Neurosurg Psychiatry.

[12] Feresiadou A, Nilsson K, Ingelsson M, Press R, Kmezic I, Nygren I, Svenningsson A, Niemela V, Gordh T, Cunningham J (2019). Measurement of sCD27 in the cerebrospinal fluid identifies patients with neuroinflammatory disease. J Neuroimmunol.

[13] Liu B, Zhong X, Lu Z, Qiu W, Hu X, Wang H (2018). Cerebrospinal fluid level of Soluble CD27 is Associated with Disease Severity in Neuromyelitis Optica Spectrum Disorder. Neuroimmunomodulation.

[14] Mescheriakova JY, Runia TF, Jafari N, Siepman TA, Hintzen RQ. Soluble CD27 Levels in Cerebrospinal Fluid as a Prognostic Biomarker in Clinically Isolated Syndrome. *JAMA Neurol* 2017, 74:286–292. 10.1001/jamaneurol.2016.4997 28055081

[15] Wong YYM, van der Vuurst RM, van Pelt ED, Ketelslegers IA, Melief MJ, Wierenga AF, Catsman-Berrevoets CE, Neuteboom RF, Hintzen RQ (2018). T-cell activation marker sCD27 is associated with clinically definite multiple sclerosis in childhood-acquired demyelinating syndromes. Mult Scler.

[16] Komori M, Blake A, Greenwood M, Lin YC, Kosa P, Ghazali D, Winokur P, Natrajan M, Wuest SC, Romm E (2015). Cerebrospinal fluid markers reveal intrathecal inflammation in progressive multiple sclerosis. Ann Neurol.

[17] Romme Christensen J, Komori M, von Essen MR, Ratzer R, Bornsen L, Bielekova B, Sellebjerg F (2019). CSF inflammatory biomarkers responsive to treatment in progressive multiple sclerosis capture residual inflammation associated with axonal damage. Mult Scler.

[18] El Mahdaoui S, Husted SR, Hansen MB, Cobanovic S, Mahler MR, Buhelt S, von Essen MR, Sellebjerg F, Romme Christensen J (2023). Cerebrospinal fluid soluble CD27 is associated with CD8(+) T cells, B cells and biomarkers of B cell activity in relapsing-remitting multiple sclerosis. J Neuroimmunol.

[19] Klein A, Selter RC, Hapfelmeier A, Berthele A, Muller-Myhsok B, Pongratz V, Gasperi C, Zimmer C, Muhlau M, Hemmer B (2019). CSF parameters associated with early MRI activity in patients with MS. Neurol Neuroimmunol Neuroinflamm.

[20] Glouchkova L, Ackermann B, Zibert A, Meisel R, Siepermann M, Janka-Schaub GE, Goebel U, Troeger A, Dilloo D (2009). The CD70/CD27 pathway is critical for stimulation of an effective cytotoxic T cell response against B cell precursor acute lymphoblastic leukemia. J Immunol.

[21] Agematsu K, Kobata T, Sugita K, Freeman GJ, Beckmann MP, Schlossman SF, Morimoto C (1994). Role of CD27 in T cell immune response. Analysis by recombinant soluble CD27. J Immunol.

[22] Huang J, Jochems C, Anderson AM, Talaie T, Jales A, Madan RA, Hodge JW, Tsang KY, Liewehr DJ, Steinberg SM (2013). Soluble CD27-pool in humans may contribute to T cell activation and tumor immunity. J Immunol.

[23] Cencioni MT, Ali R, Nicholas R, Muraro PA (2021). Defective CD19 + CD24(hi)CD38(hi) transitional B-cell function in patients with relapsing-remitting MS. Mult Scler.

[24] Hultquist JF, Hiatt J, Schumann K, McGregor MJ, Roth TL, Haas P, Doudna JA, Marson A, Krogan NJ (2019). CRISPR-Cas9 genome engineering of primary CD4(+) T cells for the interrogation of HIV-host factor interactions. Nat Protoc.

[25] Chen G, Huang AC, Zhang W, Zhang G, Wu M, Xu W, Yu Z, Yang J, Wang B, Sun H (2018). Exosomal PD-L1 contributes to immunosuppression and is associated with anti-PD-1 response. Nature.

[26] Kowal J, Arras G, Colombo M, Jouve M, Morath JP, Primdal-Bengtson B, Dingli F, Loew D, Tkach M, Thery C (2016). Proteomic comparison defines novel markers to characterize heterogeneous populations of extracellular vesicle subtypes. Proc Natl Acad Sci U S A.

[27] Magliozzi R, Fadda G, Brown RA, Bar-Or A, Howell OW, Hametner S, Marastoni D, Poli A, Nicholas R, Calabrese M (2022). Ependymal-in gradient of thalamic damage in progressive multiple sclerosis. Ann Neurol.

[28] De Groot CJ, Bergers E, Kamphorst W, Ravid R, Polman CH, Barkhof F, van der Valk P (2001). Post-mortem MRI-guided sampling of multiple sclerosis brain lesions: increased yield of active demyelinating and (p)reactive lesions. Brain.

[29] Magliozzi R, Howell O, Vora A, Serafini B, Nicholas R, Puopolo M, Reynolds R, Aloisi F (2007). Meningeal B-cell follicles in secondary progressive multiple sclerosis associate with early onset of disease and severe cortical pathology. Brain.

[30] Bankhead P, Loughrey MB, Fernandez JA, Dombrowski Y, McArt DG, Dunne PD, McQuaid S, Gray RT, Murray LJ, Coleman HG (2017). QuPath: open source software for digital pathology image analysis. Sci Rep.

[31] Howell OW, Reeves CA, Nicholas R, Carassiti D, Radotra B, Gentleman SM, Serafini B, Aloisi F, Roncaroli F, Magliozzi R, Reynolds R (2011). Meningeal inflammation is widespread and linked to cortical pathology in multiple sclerosis. Brain.

[32] Gregorutti B, Michel B, Saint-Pierre P (2017). Correlation and variable importance in random forests. Stat Comput.

[33] Ruprecht CR, Lanzavecchia A (2006). Toll-like receptor stimulation as a third signal required for activation of human naive B cells. Eur J Immunol.

[34] Krumbholz M, Theil D, Derfuss T, Rosenwald A, Schrader F, Monoranu CM, Kalled SL, Hess DM, Serafini B, Aloisi F (2005). BAFF is produced by astrocytes and up-regulated in multiple sclerosis lesions and primary central nervous system lymphoma. J Exp Med.

[35] Serafini B, Rosicarelli B, Magliozzi R, Stigliano E, Aloisi F (2004). Detection of ectopic B-cell follicles with germinal centers in the meninges of patients with secondary progressive multiple sclerosis. Brain Pathol.

[36] Ng LG, Sutherland AP, Newton R, Qian F, Cachero TG, Scott ML, Thompson JS, Wheway J, Chtanova T, Groom J (2004). B cell-activating factor belonging to the TNF family (BAFF)-R is the principal BAFF receptor facilitating BAFF costimulation of circulating T and B cells. J Immunol.

[37] Hassanshahi G, Jafarzadeh A, Esmaeilzadeh B, Arababadi MK, Yousefi H, Dickson AJ (2008). Assessment of NK cells response to hepatocyte derived chemotactic agents. Pak J Biol Sci.

[38] Blandford SN, Fudge NJ, Moore CS. CXCL10 is Associated with increased Cerebrospinal Fluid Immune Cell Infiltration and Disease Duration in multiple sclerosis. Biomolecules 2023, 13. 10.3390/biom13081204 PMC10452246 37627269

[39] Sorensen TL, Sellebjerg F, Jensen CV, Strieter RM, Ransohoff RM (2001). Chemokines CXCL10 and CCL2: differential involvement in intrathecal inflammation in multiple sclerosis. Eur J Neurol.

[40] Sorensen TL, Trebst C, Kivisakk P, Klaege KL, Majmudar A, Ravid R, Lassmann H, Olsen DB, Strieter RM, Ransohoff RM, Sellebjerg F (2002). Multiple sclerosis: a study of CXCL10 and CXCR3 co-localization in the inflamed central nervous system. J Neuroimmunol.

[41] Simpson JE, Newcombe J, Cuzner ML, Woodroofe MN (2000). Expression of the interferon-gamma-inducible chemokines IP-10 and Mig and their receptor, CXCR3, in multiple sclerosis lesions. Neuropathol Appl Neurobiol.

[42] Piccio L, Naismith RT, Trinkaus K, Klein RS, Parks BJ, Lyons JA, Cross AH (2010). Changes in B- and T-lymphocyte and chemokine levels with rituximab treatment in multiple sclerosis. Arch Neurol.

[43] Irani DN. Regulated production of CXCL13 within the Central Nervous System. J Clin Cell Immunol 2016, 7. 10.4172/2155-9899.1000460 PMC5461933 28603659

[44] Lundblad K, Zjukovskaja C, Larsson A, Cherif H, Kultima K, Burman J. CSF concentrations of CXCL13 and sCD27 before and after autologous hematopoietic stem cell transplantation for multiple sclerosis. Neurol Neuroimmunol Neuroinflamm 2023, 10. 10.1212/NXI.0000000000200135 PMC10265403 37311645

[45] Clarke CJ, Hannun YA (2006). Neutral sphingomyelinases and nSMase2: bridging the gaps. Biochim Biophys Acta.

[46] Lin WC, Lin CF, Chen CL, Chen CW, Lin YS (2011). Inhibition of neutrophil apoptosis via sphingolipid signaling in acute lung injury. J Pharmacol Exp Ther.

[47] Shamseddine AA, Airola MV, Hannun YA (2015). Roles and regulation of neutral sphingomyelinase-2 in cellular and pathological processes. Adv Biol Regul.

