# Supplementary material for: Soluble CD27 is an intrathecal biomarker of T-cell-mediated lesion activity in multiple sclerosis
Source: J Neuroinflammation. 2024 Apr 12;21:91. doi: 10.1186/s12974-024-03077-9 (PMC11015621; doi:10.1186/s12974-024-03077-9)
Supplement: Supplementary file 1 — Supplementary Material 1 [file 12974_2024_3077_MOESM1_ESM.docx]

Supplementary tables and figures.

Supplementary Table1. Main clinical and demographic characteristics of patients with MS and control cases

Supplementary Table 2. CSF Leukocyte subsets.

Supplementary Table 3. Primary and secondary antibodies are used for western blotting**.**

Supplementary Table 4. Demographic and clinical features of post-mortem MS cases

Supplementary Table 5. Demographic and clinical features of post-mortem control cases

Supplementary Table 6. Primary and secondary antibodies are used for immunohistochemistry and immunofluorescence.


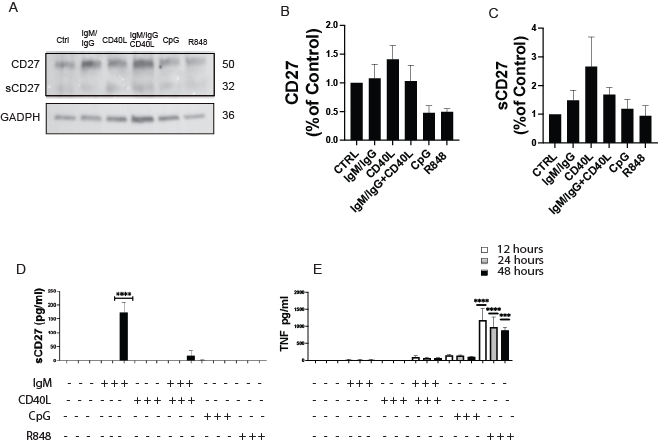


Supplementary Fig. 1. Levels of CD27 in B cells

Immunoblots (A) for CD27 in the whole cell lysate from purified B cells in resting and stimulated with anti-human IgM/IgG, (H+L) functional grade- (5ug/ml), CD40L(1ug/ml), IgM/IgG+CD40L, CpG (2.5ug/ml) and R848 (1ug/ml) for 48 hours. The protein extracts are from isolated B cells of 4 healthy donors. Quantification for (B) CD27 and (C) sCD27 as a percentage of control by western blotting. Histogram report levels of sCD27 (pg/ml) (D) and TNF (pg/ml) (E) detected on the supernatant of isolated B cells treated with IgM/IgG, CD40L, IgG/IgM +CD40L, CPG ODN and R848 at 12, 24 and 48 hours as measured by ELISA. Data show means ± SD of 3 independent experiments. Statistical analysis was performed by using ordinary 2-way ANOVA.
